# Isolation of *Enterobacter sakazakii* from Midgut of *Stomoxys calcitrans*

**DOI:** 10.3201/eid0910.030218

**Published:** 2003-10

**Authors:** Joanne V. Hamilton, Michael J. Lehane, Henk R. Braig

**Affiliations:** * University of Wales, Aberystwyth, Ceredigion, UK; † University of Wales, Bangor, Gwynedd, UK

**To the Editor:** *Enterobacter sakazakii,* a Gram-negative rod-shaped bacterium, is an emerging foodborne pathogen that can cause meningitis, sepsis, or necrotizing enterocolitis in newborns, particularly affecting premature or other immunocompromised infants. Although an environmental reservoir and mode of transmission for *E. sakazakii* has not been clearly identified, a growing number of reports suggest that powdered milk–based infant formulas can be a vehicle for infection. We report the isolation of *E. sakazakii* from the guts of larvae of the stable fly, *Stomoxys calcitrans,* demonstrating an environmental reservoir for *E. sakazakii* and raising the possibility that environmental contamination by insects may be important in the spread of this opportunistic organism.

The first two cases of neonatal meningitis caused by *E. sakazakii* were reported in the United Kingdom in 1961; both infants died (*1*). Subsequently, cases of meningitis, septicemia, necrotizing enterocolitis, brain abscesses, cerebral infarctions and dermoid cysts of the posterior fossa caused by *E. sakazakii* have been reported worldwide.

Although most documented cases involve infants, reports also describe adult infections. Adults with *E. sakazakii* infections usually have serious underlying diseases or malignancies, but adult meningitis caused by *E. sakazakii* infection has never been reported. Concern is growing regarding antibiotic resistance in *E. sakazakii*. Recently, an *E. sakazakii* wound infection in an adult patient has been reported, which was resistant to multiple antibiotics and required prolonged treatment with broad-spectrum antibiotics (*2*).

Illness and death associated with *E. sakazakii* infection in neonates vary considerably, and mortality rates as high as 80% have been recorded (*3*). Implicated infection sources include incubators, the birth canal, a blender in a milk kitchen where powdered infant formula was reconstituted and hospital environments (*E. sakazakii* growth on an agar plate in a ward specifically under infection control against multidrug-resistant *Staphylococcus aureus* [MRSA]). The presence of *E. sakazakii* in powdered infant formula has been reported often. An investigation of Enterobacteriaceae found in powdered substitutes for breast milk from 35 countries indicated that 52.2% of the 141 samples tested contained members of this family. *E. agglomerans, E. cloacae, E. sakazakii,* and *Klebsiella pneumoniae* were the most frequently isolated organisms (*4*). More recently, *E. sakazakii* has been detected in ultra–heat- treated milk, spoiled tofu, lettuce, and traditional fermented bread (khamir), and in beer mugs rinsed mechanically or in open vats. Although more vehicles for transmission are being identified, the environmental reservoir has remained elusive. A study in 1990 did not isolate this organism from samples of surface water, mud, soil, rotting wood, bird dung, grain, rodents, domestic animals, cattle, or raw cows milk (*5*). We report the isolation of *E. sakazakii* from the gut of larvae of the blood-sucking insect *Stomoxys calcitrans*.

*S. calcitrans* were reared in a laboratory culture (originally isolated from farms in North Wales, U.K.) as previously described (*6*). Larvae were removed from culture medium, washed in sterile isotonic saline solution, and surface sterilized by immersion in 100% ethanol. Larval guts were dissected aseptically and placed individually onto standard LB agar plates. Plates were incubated at 24°C overnight and were examined for the presence of bacteria the next day. Subcultures of each different colony type were streaked onto fresh LB plates until pure colonies were obtained.

A sample was taken from each pure colony and an attempt was made to identify the bacteria b using standard polymerase chain reaction (PCR) amplification of the genes encoding bacterial 16S rRNA (*7*). We identified *E. sakazakii*, *Providencia stuartii*, *Erwinia carotovora, Micrococcus luteus*, and *Serratia marcescens.* The *E. sakazakii* colony had the typical yellow pigmentation and both colony morphologic features reported for this bacterium (*3*).

The growing reports of *E. sakazakii* infections and its implied role as an emerging foodborne pathogen are of concern to clinicians, the food industry, and consumers. The high mortality rate and severity of this infection in infants, coupled with the lack of ecologic information about this organism, have fueled much debate. The voluntary product recall of a powdered infant formula by its manufacturer in 2001, after *E. sakazakii* was found in the finished product (*8*), exemplifies the need for stringent process control and the use of aseptic techniques during preparation, handling, storage, and use of reconstituted infant milk. The problem of foodborne transmission is further exacerbated by the high tolerance to heat of *E. sakazakii* (*3*). The overall risk for infection may depend on several factors, including numbers of bacteria present in the product, handling after preparation, and underlying patient characteristics.

We have identified the larval gut of the stable fly, *Stomoxys calcitrans,* as an environmental reservoir for *E. sakazakii*. Stable flies have a worldwide distribution; they are blood feeders that primarily feed on cattle, horses, dogs, pigs, and humans, but they will also take a blood meal from reptiles and birds. *Stomoxys* spp. may be found wherever cattle, pigs, or horses are kept, and more specifically, *S. calcitrans* is common in cowsheds, making contamination of milk a possibility.

The geographic distribution of *S. calcitrans* correlates well with the incidence of *E. sakazakii* infections. Both *S. calcitrans* and *E. sakazakii* infections have been reported from Denmark, the United States, Israel**,** the United Kingdom, and Germany. Additionally, *Stomoxys* spp. have a worldwide distribution and are likely present in all countries reporting *E. sakazakii* infection.

A recent study has isolated *E. sakazakii* from the guts of a laboratory colony of Mexican fruit flies, *Anastrepha ludens* (*9*). Furthermore, a technical report from a pest exterminating company in the United States records the presence of *Enterobacter sakazakii* in the house fly (*Musca domestica*), although the exact location of the bacterium on or in the fly is unclear. Insects are thus a likely major environmental reservoir of *E. sakazakii*. This conclusion suggests that in addition to stringent control measures during manufacturing and use of foodstuffs, reducing arthropod presence in hospital and manufacturing environments could result in a substantial reduction in the transmission of *E. sakazakii*. New control methods will likely need to be developed because some evidence suggests that insect traps that electrocute could play a role in the spread of infectious disease agents such as bacteria and viruses (*10*).
